# The test–retest reliability and limits of agreement of the balance evaluation systems test (BESTest) in young people with intellectual disability

**DOI:** 10.1038/s41598-023-43367-5

**Published:** 2023-09-25

**Authors:** Saeid Bahiraei, Elham Hosseini, Rahman Amiri Jomi Lou

**Affiliations:** 1 SciencesDepartment of Sport Injuries and Corrective Exercises, Faculty of Physical Education and Sport Sciences, Shahid Bahonar University of Kerman, Kerman, Iran; 2 Department of Sport Injuries and Corrective Exercises, Faculty of Physical Education and Sport Sciences, Shahid Bahonar University of Kerman, Kerman, Iran; 3 Department of Sport Injuries and Corrective Exercises, Faculty of Sports Sciences, University of Guilan, Rasht, Iran

**Keywords:** Neurological disorders, Neuroscience, Health care, Medical research, Neurology

## Abstract

Clinical tests for the assessment of postural balance in people with intellectual disability have been the most commonly used single or multi-item tests, but some tests have been developed, such as the BESTest. The purpose of the study was to evaluate the test–retest reliability and limits of agreement of the Balance Evaluation Systems Test (BESTest) in young people with intellectual disabilities. A descriptive cross-sectional study was conducted with 65 young people (ages 16–25 years) with intellectual disability. The participants completed the BESTest (27 items) twice. Intraclass correlation coefficients (ICC), 95% confidence intervals (CIs), and standard error of measurement (SEM) were calculated to determine the test–retest reliability of the BESTest. The BESTest overall scores' test–retest reliability was rated as excellent (≥ 0.75). Stability limits/verticality and reactive are fair to good (≥ 0.40– < 0.75). Biomechanical constraints, transitions and anticipatory movements, sensory orientation, and gait stability were excellent (≥ 0.75). Current evidence shows that young people with intellectual disabilities have impaired postural balance. However, there appears to be a lack of assessment tools that reliably evaluate the postural balance of this population. The results from this investigation show that BESTest provides "excellent reliability" (≥ 0.75) to assess postural balance in young people with intellectual disability.

## Introduction

Impairments in both intellectual and adaptive functioning behaviors characterize intellectual disability (ID), which is a type of neurodevelopmental disorder^1^. Adaptive behavior is defined as the conceptual, social and practical skills involving tasks performed by persons in their everyday lives^2^. Young people with ID often exhibit delays in maturation and motor growth to varying degrees, which can limit their functional abilities. Postural control is one motor skill in which people with ID tend to experience more limitations. Postural control is one motor skill in which people with ID tend to experience more limitations^3,4^. This phenomenon can be explained by the physiopathology of ID, which usually involves some degree of incomplete development of the central nervous system (CNS), which controls motor and cognitive functions^5^. These people age prematurely, and as with healthy people, their postural control deteriorates with age because of the decline of the various postural control subsystems (primarily the somatoaesthetic, vestibular, and visual subsystems)^6^. Studies have shown that gait deceleration can be an early indicator of balance decline^7,8^. Various evaluation tools for motor development and skill are commonly used in young people with ID.

The clinical tests for the evaluation of balance and gait that have been most commonly used in this population are: the Berg Balance Scale (BBS)^9^, the Tinetti Scale^3^, Single-leg Stance^10^, the Functional Reach Test (FRT)^10,11^, the Lateral Reach Test (LRT)^10,11^, the Biodex Balance System (BBS)^12,13^, the Force Plate and the Timed Up and Go (TUG)^10,11^. These functional balance scales are useful in reporting balance disorders, but they cannot identify the type of disorders underlying balance disorders. However, performance balance tests usually examine the performance in a series of tests; in evaluating the functional balance, single or multi-item tests are usually applied^14^. Tests including items such as tandem standing and standing on one foot provide less information about balance disorders. Multi-item tests can provide much more useful information^15^. Postural control will no longer be one system or a group of balance and postural reflexes. Postural control is complicated skill that comprises a compromise between sensorimotor processes^16^. According to these cases, the Balance Evaluation Systems Test (BESTest) has recently been developed based on a conceptual model of balance control in which six different systems or domains contribute to balance control. The BESTest evaluates the following systems: biomechanical constraints, stability limits, and verticality, anticipatory postural adjustments, automatic postural responses, sensory organization, and gait stability^2,17^.

This tool is a special assessment for any age and severity of Parkinson’s disease, cerebral ataxia disorders, vestibular disorders, neuropathy, brain damage, multiple sclerosis, brain stroke, cerebral palsy, intellectual and recognition disorders (ID and Down syndrome), and other balance disorders^18–20^. Nevertheless, previous studies have primarily concentrated on particular aspects of postural control, as the comprehensive clinical evaluation necessary for individuals with intellectual disabilities has limited the scope of research. For example, in children with a nervous disorder, measuring probable disorders in the systems involved with postural control is important^21^. For example, children with cerebral palsy have demonstrated disorders in anticipatory mechanisms (feed-forward postural adjustments), musculoskeletal systems (muscular strength and the joint range of motion required for static balance), and sensory systems (visual functions and proprioception). This study shows how a population can experience a wide range of postural control conditions and how a lack of comprehensive clinical evaluation exists for people with ID. Out of the present clinical evaluations for measuring model postural disorders in people, none evaluates all the systems involved with postural control, and a large amount of the data is limited^19,22^. Accordingly, the test–retest reliability of the BESTest for young people with ID remains unclear. Thus, the investigation evaluated the test–retest reliability of the BESTest for measuring postural balance in young people with ID.

## Methods

### Participants

Sixty-five young people with ID (25 females and 40 males) aged 16 to 25 years volunteered to participate in the investigation. ID with IQs ranging from 50 to 70 were chosen from intellectual disability centers and special education schools in the provinces of Guilan and Ardabil (Table 1). The inclusion criteria were (a) age 16–25 years; (b) a diagnosis of ID defined by a full-scale IQ < 70 obtained on an individually administered test of intelligence, and (c) being able to follow simple commands cognitively. Individuals with coexisting neurological or cardiovascular impairments were excluded. Individuals who had received any physical or exercise training during the study period were also excluded.

**Table 1:** Descriptive characteristics (*n* = 65; mean + SD; range).

Characteristics	Mean + SD; (range)
Age (years)	21.20 ± 4.81(16–25)
Weight (kg)	65.73 ± 15.86(41.60–97.50)
Height (cm)	161.13 ± 8.34(151–176)
BMI (kg/m^2^)	25.12 ± 4.79(18.01–31.84)
Male	61%
Female	39%
IQ	50–70

### Procedures

All participants came to the research laboratory for three visits. During the first visit, a detailed explanation of the study protocol was provided to the participants and their parents and/or educators. After the explanation of the study, parents and educators provided informed satisfaction, and the participants signed an agreement. Anthropometric assessments of the height and weight of each participant were completed. The participants reported their children's date of birth and IQ, and they completed the BESTest. Only on the second and third visits did the participants complete the BESTest. We separated all visits by at least two weeks to account for any training effect. No intervention or other testing by the current researchers was implemented between visits. The participant's parents or guardians did not report any other intervention or testing during this time, and participants were encouraged to maintain their typical activity behaviors. Before each assessment, a trained researcher performed a demonstration.

### Ethics statement and consent to participate

This study was approved by the Ethics Review Committee of the Guilan University of Medical Sciences and all participants signed informed consent (No. IR. GUMS.REC.1397.021) and follows the guidelines of the Declaration of Helsinki 2013.

### Balance Evaluation Systems Test (BESTest)

BESTest is a quantitative evaluation method aimed at detecting faulty systems that underpin postural control, which is responsible for poor functional balance. BESTest was developed in 2009 and consists of 27 activities (items) that are divided into six categories. These categories show how well a particular balancing control system works, such as biomechanical constraints, stability limits or verticality, anticipatory postural adjustments, postural responses, sensory orientation, and gait stability. Each item can receive a maximum score of 15 to 21, and every component in the BESTest is rated between 0 (lowest performance) and 3 (highest performance). The maximum total score was 108 points. Each examinee must complete all the tests within 30 minutes^19^. Which balance control system is malfunctioning may be determined, which helps to target the therapy. The Activities-specific Balance Confidence Scale (ABC Scale) has been used to assess concurrent validity (r = 0/636, p = 0.01). The ABC Scale measures a person's level of assurance that they will not lose their balance while conducting 16 activities of daily living. The ABC Scale has a score range of 0 to 100, with 0 indicating no confidence and 100 showing complete confidence in the person's ability to accomplish the task without losing balance. Also, the examination durability was 0.91, while each system durability ranged from 0.79 to 0.96^18,23^. Please refer to Supplementary Material (Appendix 1) for further details regarding the instructions for the test, the required tools, and the performance.

### Statistical analysis

Data were analyzed using SPSS version 22. Participants' descriptive characteristics are presented as the mean standard deviation (SD). Because all participants completed each assessment in the BESTest, data from all participants (n = 65) were analyzed. The best score for each trial of the assessments was used in the analysis. To determine the test–retest reliability of the BESTest, intraclass correlation coefficients (ICCs) and 95% confidence intervals (CIs) were calculated. A two-way mixed model approach, mean rating of 1 (k = 1), and absolute agreement was used^24^. Absolute agreement was introduced by Stine (1989). Observers are in agreement if their scores differ by a constant or if they are a fixed linear function of each other. The limit of agreement estimates observer differences. The agreement is satisfactory if the absolute limit (observer differences) is below an acceptable difference^25^. A reliable statistic that can be used for both intrarater and interrater reliability testing is Cohen's kappa. Similar to correlation coefficients, it can be in the range of 0 to 1, where 0 denotes the degree of agreement that would be predicted by chance and 1 denotes perfect agreement between the raters. The kappa is a standardized value that is interpreted consistently across numerous studies, similar to all correlation statistics. Cohen recommended that the Kappa result be interpreted as follows: values 0 denote no agreement, 0.01–0.20 indicate no to little agreement, 0.21–0.40 indicate reasonable agreement, 0.41- 0.60 indicate moderate agreement, 0.61–0.80 indicate substantial agreement, and 0.81–1.00 indicate perfect agreement. However, this interpretation permits a very low level of inter-rater agreement to be referred to as "substantial"^26^. The formula (SEM = SD_1st Test_ × √(1 − ICC)) was used to calculate the standard error of measurement (SEM). These statistics indicate the errors associated with specific assessments. We then calculated the minimal detectable change (MDC) based on the SEM as follows: MDC = 1.96 × SEM × √2. Furthermore, the Bland and Altman approach was used to determine the level of discrepancy between test and retest measurements. This technique involves creating a scatter plot that depicts the differences between the two sets of values against their average. The method also employs the concept of Limits of Agreement (LOA), which indicates the average difference between the two tests and its 95% confidence interval (CI)^27^. Bland–Altman calculations were performed using Graph Pad Prism (Version 6; GraphPad Software Inc). Significance was set at an alpha level of *P* < 0.05.

## Results

Fifteen young people participated in the study. Demographic information for the participants is presented in Table 1. BEST overall scores and test–retest reliability were rated excellent (ICC = 0.92, 95% CI 0.87 to 0.95, *p* < 0.001, SEM = 2.48, MDC: 6.87). The test–retest reliability results are presented for each of the subtests in Table 2. Eight assessments were considered to have “fair to good” reliability (stability limits/verticality and reactive) (n = 8), and the remaining assessments (biomechanical constraints, transitions/anticipatory, sensory orientation, and stability in gait) had excellent reliability (n = 19) (Table 2). The SEM for the subtest assessments ranged from 1.95 to 7.23; specific values for the individual assessments are included in Table 2. The MDC for the subtest assessments ranged from 5.40 to 20.04; specific values for the individual assessments are included in Table 2.

**Table 2:** BESTest subtest percent scores, intraclass correlations (95% CI), standard error of the measurement, minimal detectable change,and subtest scores (N = 65).

BESTest subtest	Trials 1 Mean ± SD; (Range)	Trials 2 Mean ± SD; (Range)	Trials 3 Mean ± SD; (Range)	ICC (95% CI)	SEM	MDC	Classification of subtest ICCs	No. of Subtests (%)	Absolute limits (95%)
Biomechanical constraints									
Base of Support COM Alignment Ankle Strength & Range Hip/Trunk Lateral Strength Sit on Floor and Stand Up	59.18 ± 11.27; (46.67–80)	59.10 ± 11.54; (46.67–80)	58.36 ± 11.79; (46.67–80)	0.97 (0.98 to 0.96)	1.95	5.40	Excellent reliability (≥ 0.75)	5/27(18.52%)	0.10 (− 8.06 to 8.26)
Stability Limits/Verticality									
Sitting Verticality and Lateral Lean Functional Reach Forward Functional Reach Lateral	69.67 ± 9.91; (47.62–85.71)	69.89 ± 10.28; (47.62–85.71)	69.81 ± 7.91; (47.62–85.71)	0.69 (0.54 to 0.81)	5.52	15.30	Fair to good reliability (≥ 0.40– < 0.75)	3/27(11.11%)	− 0.22 (− 21.50 to 21.07)
Transitions/anticipatory									
Sit to Stand Rise to Toes Stand on One Leg Alternate Stair Touching Standing Arm Raise	71.37 ± 7.75; (55.56–88.89)	71.62 ± 8.84; (71.62–83.33)	74.19 ± 8.12; (61.11–83.33)	0.85 (0.77 to 0.90)	2.99	8.29	Excellent reliability (≥ 0.75)	5/27(18.52%)	− 0.26 (− 12.34 to 11.83)
Reactive									
In Place Response Forward In Place Response—Backward Compensatory Stepping Correction- Forward Compensatory Stepping Correction—Backward Compensatory Stepping Correction -lateral	60.34 ± 12.22; (33.33–83.33)	63.08 ± 12.44; (33.33–88.89)	62.31 ± 12.90; (33.33–83.33)	0.65 (0.47 to 0.77)	7.23	20.04	Fair to good reliability (≥ 0.40– < 0.75)	5/27(18.52%)	− 2.73 (− 30.77 to 25.30)
Sensory orientation									
Sensory Integration for Balance (Modified CTSIB) Incline Eyes Closed	76.31 ± 8.42; (60.00–86.67)	77.84 ± 7.91; (60.00–86.67)	77.33 ± 8.15; (60.00–86.67)	0.85 (0.77 to 0.90)	3.26	9.04	Excellent reliability (≥ 0.75)	2/27(7.40%)	− 1.54 (− 16.65 to 13.57)
Stability in Gait									
Gait – Level Surface Change in Gait Speed Walk with Head Turns – Horizontal Walk with Pivot Turns Step over Obstacles Timed “Get UP & GO” Timed “Get Up & Go” With Dual Task	57.10 ± 7.64; (47.62–71.43)	57.73 ± 7.64; (47.62–71.43)	58.02 ± 6.46; (47.62–71.43)	0.82 (0.73 to 0.89)	3.24	8.98	Excellent reliability (≥ 0.75)	7/27(25.92%)	− 0.66 (− 14.25 to 12.93)
Percent Total Score	65.41 ± 8.76; (49.07–82.41)	66.28 ± 6.06; (53.70–77.78)	66.45 ± 5.94; (52.78–77.78)	0.92 (0.87 to 0.95)	2.48	6.87	Excellent reliability (≥ 0.75)	27/27(100%)	− 0.87 (− 10.46 to 8.72)

*ICC* intraclass correlation coefficient, *MDC* minimal detectable change, *SEM* standard error of measurement agreement.

**Figure 1:**
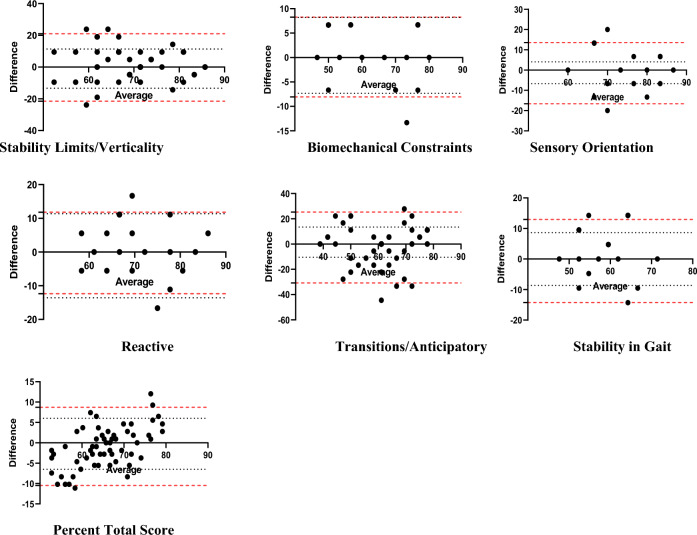
Test–retest agreement for the BESTest index scores expressed by Bland and Altman plots. The solid line is the mean of the difference in all subjects; the dotted lines define LOA are mean of the difference 1.96 SD. (Percent Total Score, Stability in Gait, Sensory Orientation, Reactive, Transitions/Anticipatory, Stability Limits/Verticality, Biomechanical Constraints.)

Figure 1 shows the differences between the test and retest plotted against their means for each subject with 95% CI and 95% Limits of Agreement (LOA). The systematic errors (mean difference between test and retest) for the analyzed test were nearly zero, and the 95% limits of agreement were narrow, indicating good reliability of the measurement.

## Discussion

This study aimed to determine the test–retest reliability of BESTest in young people with ID. Overall, BESTest appeared to be a reliable test for assessing postural control in young people with ID for epidemiological research aimed at determining the effects of interventions designed to improve physical function in this population. To the authors’ knowledge, no studies have considered the test–retest reliability of the BESTest in young people with ID. Postural control tests that have low or otherwise senseless reliability coefficients should not be used to assess postural control in people with ID. Given that existing tools for assessing clinical balance cannot assist the therapist in diagnosing the underlying causes of balance disorder, BESTest can distinguish balance subsystems and can purposefully plan the treatment process^23^. Several statistical methods and indices have been proposed to test the reliability of outcome assessment; however, it was decided that both relative and absolute reliability should be introduced^28^. For relative reliability, the ICC is the most used statistical method because it shows the level of agreement among tests. For absolute reliability, the SEM can reflect the reliability of the response with 95% CI, which provides useful information relevant to the actual value of real change^29^.

Six subsystems underlying the control of balance are targeted in BESTest regarding body position and motion with the ability to generate forces to control body position. Each system consists of neurophysiological mechanisms that control a particular aspect of postural control. The validity and reliability of BESTest have been investigated in several studies, including a variety of patient populations with balance-related disorders. For example, Lampropoulou et al.and Charlotte S.L et al., revealed that it is a valid and reliable balance evaluation method for chronic stroke patients that demonstrates stability, repeatability, and good distribution. Mini-BESTest is the best scientific tool for measuring balance in chronic stroke because of its high reliability and validity. Godi et al., The Berg scale and Mini-BESTest demonstrate similar results, although the Mini-BESTest has a smaller ceiling effect, somewhat higher reliability, and more accuracy in classifying patients with considerable balance function improvement. In addition, Lofgren et al. indicated that MiniBESTest is able to differentiate between individuals with mild and moderate Parkinson’s disease; however, when used in clinical balance evaluations, the large measurement error must be considered. Overall, these studies provide ICC values for total scores ranging from.80 to.97, measurement errors ranging from and MDC ranging from 2.4 to 5.2^30–35^. In our study, excellent reliability was found for both the biomechanical constraints, transitions and anticipatory, sensory orientation, and stability in gait sections, which is consistent with these findings in other various populations, such as people with stroke, Parkinson’s disease, spinal cord injury, and multiple sclerosis, and also in people with balance disorders and people with an increased risk of falling.

On the other hand, studies such as Villamonte et al. measured test–retest reliability scores on 16 balance tests in children, teenagers, and young adults with ID. Among the tests conducted are the STS (20 s long) and the TUG (9 m distance), the former being reliable (ICC > 50) only in young women and young men, while the latter is not reliable for any group of ID. Among the limitations of the study are its small sample (21 people) and very varied age range (5–31 years old)^36^. PH Boer and S.J. Moss explored the test–retest reliability and minimal detectable change of selected functional fitness test items in adults with ID. The results indicated that all tests showed excellent results (ICCs > 0.9). All SEM values demonstrated acceptable measurement precision (SEM < SD/2). Values for MDC90 are provided for all 12 tests^37^. The Consensus-Based Standards for the Selection of Health Measurement Instruments initiative defines domain reliability as "the degree to which the measurement is free from measurement error," which implies that scores for unchanged patients are constant in repeated measurements. Estimations of the MDC and limits of agreement are important to define the change and the boundaries that need to be exceeded to show a change beyond the measurement error, i.e., a true change. The current study findings indicate that a change of 10 points or more in the BESTest total score is required to determine a true change in balance control in ID. However, if the sample consists of only ID, a change of 10 points or more is needed.

The results of this investigation suggest that BESTest is a reliable test that can be used to assess overall postural control in young people with ID. In this study, the kappa values for all items except items 2 and 4 were considered excellent. The kappa value for items 2 and 4 included in the section on stability limits/verticality and reactive postural control was fair to good. This contributed to the lower reliability found in this section compared with the other BESTest sections. The assessment of items 2 and 4 on the first test occasion seemingly caused a learning effect that biased the assessment on the second test occasion; that is, the learning effect caused anticipation when the item was reassessed, which made the performance of the item less reactive and more proactive^35^.

## Conclusion

The results of this study suggest that the reliability of the BESTest has been confirmed in young people with ID, and it introduces it as a suitable tool for assessing overall postural control in young people with ID. Verifying assessments is critical for researchers and medical professionals because maintaining balance affects physical activity and health of individuals with ID. Therefore, BESTest can be introduced as a suitable and valid assessment test for young people with ID.

## Limitations

This study has several strengths, including the use of a single researcher to collect all the data to ensure the consistency of the assessments. Nevertheless, there are some limitations to this study. First, results from this study are valid for individuals with ID; they cannot be generalized to subjects with other sub-types of ID. Also, the study acknowledges potential limitations in the BESTest (Balance Evaluation Systems Test) interpretation, including interference from other disabilities or impairments. Participants with varying motor or cognitive impairments could introduce confounding factors, affecting test outcomes. The study emphasizes the importance of participant information, including their IQ scores and broader scope of disabilities, to provide a more comprehensive understanding of the results in future research. Second, data were not collected on socioeconomic status or sports that the participants practiced. These factors may influence postural control levels. The average age of the participants was 21.1 years, which may have influenced postural control as older young people may have performed better on the BESTest when compared to adults and individuals.

## Future research

In the future, research should concentrate on people who have varying degrees of intellectual disability or who are of different ages in order to study the ability of the scale to differentiate between people of different ages or between people who have varying degrees of the severity of the illness. In addition, in order to properly establish the scale in clinical practice, it is of vital necessity to conduct psychometric evaluations of the scale on patients who have balance impairments because of various neurological diseases. A meaningful clinical change will also give clinicians useful information regarding the effectiveness of a treatment protocol in enhancing the balance ability of patients who are recovering from a variety of diseases at varying stages.

### Supplementary Information

Supplementary Information.

## Data Availability

The datasets generated and analyzed during the current study are not publicly available due to privacy and restrictions, but are available from the corresponding author upon reasonable request.
