# Supplementary material for: The test–retest reliability and limits of agreement of the balance evaluation systems test (BESTest) in young people with intellectual disability
Source: Sci Rep. 2023 Sep 25;13:15968. doi: 10.1038/s41598-023-43367-5 (PMC10520014; doi:10.1038/s41598-023-43367-5)
Supplement: Supplementary file 1 — Supplementary Information. [file 41598_2023_43367_MOESM1_ESM.pdf]

BESTest

Balance Evaluation – Systems Test

Fay Horak PhD Copyright 2008

TEST NUMBER/SUBJECT CODE ____________________ **DATE** ____________________

EXAMINER NAME ____________________

EXAMINER Instructions for BESTest

1. Subjects should be tested with flat heeled shoes or with shoes and socks off.
2. If subject must use an assistive device for an item, score that item one category lower

Tools Required

- Stop watch
- Measuring tape mounted on wall for Functional Reach test
- Approximately 60 cm x 60 cm (2 X 2 ft) block of 4-inch, medium-density, Tempur® foam
- 10 degree incline ramp (at least 2 x 2 ft) to stand on
- Stair step, 15 cm (6 inches) in height for alternate stair tap
- 2 stacked shoe boxes for obstacle during gait
- 2.5 Kg (5-lb) free weight for rapid arm raise
- Firm chair with arms with 3 meters in front marked with tape for Get Up and Go test
- Masking tape to mark 3 m and 6 m lengths on the floor for Get Up and Go

SUMMARY OF PERFORMANCE: CALCULATE PERCENT SCORE

**Section I:**	________/15 x 100 =	_______	**Biomechanical Constraints**
**Section II:**	________/21 x 100 =	_______	**Stability Limits/Verticality**
**Section III:**	________/18 x 100 =	_______	**Transitions/Anticipatory**
**Section IV**	________/18 x 100 =	_______	**Reactive**
**Section V:**	________/15 x 100 =	_______	**Sensory Orientation**
**Section VI:**	________/21 x 100 =	_______	**Stability in Gait**
**TOTAL:**	________/108 points =	________	**Percent Total Score**

BESTest- Inter-rater Reliability
Balance Evaluation – Systems Test

Subjects should be tested with flat heeled shoes or shoes and socks off. If subject must use an assistive device for an item, score that item one category lower. If subject requires physical assistance to perform an item score the lowest category (0) for that item.

I. BIOMECHANICAL CONSTRAINTS **SECTION I: ________/15 POINTS**

1. BASE OF SUPPORT
   - (3) Normal: Both feet have normal base of support with no deformities or pain
   - (2) One foot has deformities and/or pain
   - (1) Both feet has deformities OR pain
   - (0) Both feet have deformities AND pain

2. COM ALIGNMENT
   - (3) Normal AP and ML CoM alignment and normal segmental postural alignment
   - (2) Abnormal AP OR ML CoM alignment OR abnormal segmental postural alignment
   - (1) Abnormal AP OR ML CoM alignment AND abnormal segmental postural alignment
   - (0) Abnormal AP AND ML CoM alignment

3. ANKLE STRENGTH & RANGE
   - (3) Normal: Able to stand on toes with maximal height and to stand on heels with front of feet up
   - (2) Impairment in either foot of either ankle flexors or extensors (i.e. less than maximum height)
   - (1) Impairment in two ankle groups (eg; bilateral flexors or both ankle flexors and extensors in 1 foot)
   - (0) Both flexors and extensors in both left and right ankles impaired (i.e. less than maximum height)

4. HIP/TRUNK LATERAL STRENGTH
   - (3) Normal: Abducts both hips to lift the foot off the floor for 10 s while keeping trunk vertical
   - (2) Mild: Abducts both hips to lift the foot off the floor for 10 s but without keeping trunk vertical
   - (1) Moderate: Abducts only one hip off the floor for 10 s with vertical trunk
   - (0) Severe: Cannot abduct either hip to lift a foot off the floor for 10 s with trunk vertical or without vertical

5. SIT ON FLOOR AND STANDUP *Time_____________ secs*
   - (3) Normal: Independently sits on the floor and stands up
   - (2) Mild: Uses a chair to sit on floor OR to stand up
   - (1) Moderate: Uses a chair to sit on floor AND to stand up
   - (0) Severe: Cannot sit on floor or stand up, even with a chair, or refuses

II. STABILITY LIMITS **SECTION II: ________/21 POINTS**

6. SITTING VERTICALITY AND LATERAL LEAN

Lean: Left	Lean: Right	Lean	Verticality: Left	Verticality: Right	Verticality
(3)	(3)	Maximum lean, subject moves upper shoulders beyond body midline, very stable	(3)	(3)	Realigns to vertical with very SMALL or no OVERSHOOT
(2)	(2)	Moderate lean, subject's upper shoulder approaches body midline or some instability	(2)	(2)	Significantly Over- or under-shoots but eventually realigns to vertical
(1)	(1)	Very little lean, or significant instability	(1)	(1)	Failure to realign to vertical
(0)	(0)	No lean or falls (exceeds limits)	(0)	(0)	Falls with the eyes closed

7. FUNCTIONAL REACH FORWARD *Distance reached: ______ cm OR_________inches*

(3) Maximum to limits: >32 cm (12.5 in)
(2) Moderate: 16.5 cm - 32 cm (6.5 – 12.5 in)
(1) Poor: < 16.5 cm (6.5 in)
(0) No measurable lean – or must be caught

8. FUNCTIONAL REACH LATERAL *Distance reached: Left _____ cm (_____in) Right _____ cm (_____in)*

Left	Right	
(3)	(3)	Maximum to limit: > 25.5 cm (10 in)
(2)	(2)	Moderate: 10-25.5 cm (4-10 in)
(1)	(1)	Poor: < 10 cm (4 in)
(0)	(0)	No measurable lean, or must be caught

III. TRANSITIONS- ANTICIPATORY POSTURAL ADJUSTMENT SECTION III. ____________/18 POINTS

9. SIT TO STAND

(3) Normal: Comes to stand without the use of hands and stabilizes independently
(2) Comes to stand on the first attempt with the use of hands
(1) Comes to stand after several attempts or requires minimal assist to stand or stabilize or requires touch of back of leg or chair
(0) Requires moderate or maximal assist to stand

10. RISE TO TOES

(3) Normal: Stable for 3 sec with good height
(2) Heels up, but not full range (smaller than when holding hands so no balance requirement) -OR- slight instability & holds for 3 sec
(1) Holds for less than 3 sec
(0) Unable

11. STAND ON ONE LEG

Left *Time in Sec:____________*	Right *Time in Sec: ___________*
(3) Normal: Stable for > 20 s	(3) Normal: Stable for > 20s
(2) Trunk motion, OR 10-20 s	(2) Trunk motion, OR 10-20 s
(1) Stands 2-10 s	(1) Stands 2-10s
(0) Unable	(0) Unable

12. ALTERNATE STAIR TOUCHING *# of successful steps: ________ Time in seconds: _________*

(3) Normal: Stands independently and safely and completes 8 steps in < 10 seconds
(2) Completes 8 steps (10-20 seconds) AND/OR show instability such as inconsistent foot placement, excessive trunk motion, hesitation or arhythmical
(1) Completes < 8 steps – without minimal assistance (i.e. assistive device) OR > 20 sec for 8 steps
(0) Completes < 8 steps, even with assistive devise

13. STANDING ARM RAISE

(3) Normal: Remains stable
(2) Visible sway
(1) Steps to regain equilibrium/unable to move quickly w/o losing balance
(0) Unable, or needs assistance for stability

IV. REACTIVE POSTURAL RESPONSE **SECTION IV: ________/18 POINTS**

14. IN PLACE RESPONSE- FORWARD
(3) Recovers stability with ankles, no added arms or hips motion
(2) Recovers stability with arm or hip motion
(1) Takes a step to recover stability
(0) Would fall if not caught OR requires assist OR will not attempt

15. IN PLACE RESPONSE- BACKWARD
(3) Recovers stability at ankles, no added arm / hip motion
(2) Recovers stability with some arm or hip motion
(1) Takes a step to recover stability
(0) Would fall if not caught -OR- requires assistance -OR- will not attempt

16. COMPENSATORY STEPPING CORRECTION- FORWARD
(3) Recovers independently a single, large step (second realignment step is allowed)
(2) More than one step used to recover equilibrium, but recovers stability independently OR 1 step with imbalance
(1) Takes multiple steps to recover equilibrium, or needs minimum assistance to prevent a fall
(0) No step, OR would fall if not caught, OR falls spontaneously

17. COMPENSATORY STEPPING CORRECTION- BACKWARD
(3) Recovers independently a single, large step
(2) More than one step used, but stable and recovers independently OR 1 step with imbalance
(1) Takes several steps to recover equilibrium, or needs minimum assistance
(0) No step, OR would fall if not caught, OR falls spontaneously

18. COMPENSATORY STEPPING CORRECTION- LATERAL

Left	Right
(3) Recovers independently with 1 step of normal length/width (crossover or lateral OK)	(3) Recovers independently with 1 step of normal length/width (crossover or lateral OK)
(2) Several steps used, but recovers independently	(2) Several steps used, but recovers independently
(1) Steps, but needs to be assisted to prevent a fall	(1) Steps, but needs to be assisted to prevent a fall
(0) Falls, or cannot step	(0) Falls, or cannot step

V. SENSORY ORIENTATION **SECTION V: ________/15 POINTS**

19. SENSORY INTEGRATION FOR BALANCE (MODIFIED CTSIB)

**A -EYES OPEN, FIRM SURFACE**	**B -EYES CLOSED, FIRM SURFACE**	**C -EYES OPEN, FOAM SURFACE**	**D -EYES CLOSED, FOAM SURFACE**
Trial 1 ______sec	Trial 1 ______sec	Trial 1 _______sec	Trial 1______sec
Trial 2 ______sec	Trial 2 _____sec	Trial 2 ______sec	Trial 2 ______sec
(3) 30s stable	(3) 30s stable	(3) 30s stable	(3) 30s stable
(2) 30s unstable	(2) 30s unstable	(2) 30s unstable	(2) 30s unstable
(1) < 30s	(1) < 30s	(1) < 30s	(1) < 30s
(0) Unable	(0) Unable	(0) Unable	(0) Unable

20. INCLINE- EYES CLOSED
Toes Up
(3) Stands independently, steady without excessive sway, holds 30 sec, and aligns with gravity
(2) Stands independently 30 SEC with greater sway than in item 19B -OR- aligns with surface
(1) Requires touch assist -OR- stands without assist for 10-20 sec
(0) Unable to stand >10 sec -OR- will not attempt independent stance

VI. STABILITY IN GAIT **SECTION V: ________/21 POINTS**

21. GAIT – LEVEL SURFACE *Time________secs.*
    - (3) Normal: walks 20 ft., good speed (≤ 5.5 sec), no evidence of imbalance.
    - (2) Mild: 20 ft., slower speed (>5.5 sec), no evidence of imbalance.
    - (1) Moderate: walks 20 ft., evidence of imbalance (wide-base, lateral trunk motion, inconsistent step path) – at any preferred speed.
    - (0) Severe: cannot walk 20 ft. without assistance, or severe gait deviations OR severe imbalance

22. CHANGE IN GAIT SPEED
    - (3) Normal: Significantly changes walking speed without imbalance
    - (2) Mild: Unable to change walking speed without imbalance
    - (1) Moderate: Changes walking speed but with signs of imbalance,
    - (0) Severe: Unable to achieve significant change in speed AND signs of imbalance

23. WALK WITH HEAD TURNS – HORIZONTAL
    - (3) Normal: performs head turns with no change in gait speed and good balance
    - (2) Mild: performs head turns smoothly with reduction in gait speed,
    - (1) Moderate: performs head turns with imbalance
    - (0) Severe: performs head turns with reduced speed AND imbalance AND/OR will not move head within available range while walking.

24. WALK WITH PIVOT TURNS
    - (3) Normal: Turns with feet close, FAST (≤ 3 steps) with good balance.
    - (2) Mild: Turns with feet close SLOW (≥4 steps) with good balance
    - (1) Moderate: Turns with feet close at any speed with mild signs of imbalance
    - (0) Severe: Cannot turn with feet close at any speed and significant imbalance.

25. STEP OVER OBSTACLES *Time________sec*
    - (3) Normal: able to step over 2 stacked shoe boxes without changing speed and with good balance
    - (2) Mild: steps over 2 stacked shoe boxes but slows down, with good balance
    - (1) Moderate: steps over shoe boxes with imbalance or touches box.
    - (0) Severe: cannot step over shoe boxes AND slows down with imbalance or cannot perform with assistance.

26. TIMED "GET UP & GO" *Get Up & Go: Time _____________sec*
    - (3) Normal: Fast (<11 sec) with good balance
    - (2) Mild: Slow (>11 sec with good balance)
    - (1) Moderate: Fast (<11 sec) with imbalance.
    - (0) Severe: Slow (>11 sec) AND imbalance.

27. Timed "Get Up & Go" With Dual Task *Dual Task: Time ________________sec*
    - (3) Normal: No noticeable change between sitting and standing in the rate or accuracy of backwards counting and no change in gait speed.
    - (2) Mild: Noticeable slowing, hesitation or errors in counting backwards OR slow walking (10%) in dual task
    - (1) Moderate: Affects on BOTH the cognitive task AND slow walking (>10%) in dual task.
    - (0) Severe: Can't count backward while walking or stops walking while talking

INSTRUCTIONS FOR BESTEST

BIOMECHANICAL CONSTRAINTS

1. BASE OF SUPPORT

Examiner Instructions: Closely examine both feet to look for deformities or complaints of pain such as abnormal pronation/supination, abnormal or missing toes, pain from plantar fascitis, bursitis, etc).

Patient: Stand up in your bare feet and tell me if you currently have any pain in your feet or ankles or legs.

2. COM ALIGNMENT

Examiner Instructions: Look at the patient from the side and imagine a vertical line through their center of body mass (CoM) to their feet. (The CoM is the imaginary point inside or outside the body about which the body would rotate if floating in outer-space.) In an adult, standing erect, a vertical line through the CoM to the support surface is aligned in front of the vertebrae at the umbilicus and passes about 2 cm in front of the lateral malleolus, centered between the two feet. Abnormal segmental postural alignment such as scoliosis or kyphosis or asymmetries may or may not affect CoM alignment.

Patient: Stand relaxed, looking straight ahead

3. ANKLE STRENGTH & RANGE

Examiner Instructions: Ask the patient rest their fingertips in your hands for support while they stand on their toes has high as possible and then stand on their heels. Watch for height of heel and toe lift.

Patient*:* Rest your fingers in my hands for support while you stand on your toes**.** Now stand on your heels by lifting up your toes. Maintain each position for 3 sec.

4. HIP/TRUNK LATERAL STRENGTH

Examiner Instructions: Ask the patient to rest their fingertips in your hands while they lift their leg to the side off the floor and hold. Count for 10 sec while their foot is off the floor with a straight knee. If they must use moderate force on your hands to keep their trunk upright, score as without keeping trunk vertical.

Patient: Lightly rest your fingertips in my hands while you lift your leg out to the side and hold until I tell you to stop. Try to keep your trunk vertical while you hold your leg out.

5. SIT ON FLOOR AND STANDUP

Examiner Instructions: Start with the patient standing near a sturdy chair. The patient can be considered to be sitting when both buttocks are on the floor. If the task takes more than 2 minutes to complete the task, with or without a chair, score 0. If the patient requires any physical assistance, score 0.

Patient: Are you able to sit on the floor and then stand up, in less than 2 minutes? If you need to use a chair to help you go onto the floor or to stand up, go ahead but your score will be affected. Let me know if you cannot sit on the floor or stand up without my help.

SITTING STABILITY LIMITS

6. VERTICALITY AND LATERAL LEAN

Examiner Instructions: Patient is sitting comfortably on a firm, level, armless surface (bench or chair) with feet flat on floor. It is okay to lift ischium or feet when leaning. Watch to see if the patient returns to vertical smoothly without over or undershooting. Score the worst performance to each side.

Patient: Cross your arms over your chest. Place feet shoulder width apart. I'll be asking you to close your eyes and lean to one side as far as you can. You'll keep your spine straight, and lean sideways as far as you can without losing your balance OR using your hands. Keeping your eyes closed, return to your starting position when you've leaned as far as you can. It's okay to lift your buttocks and feet. Close your eyes Lean now. (REPEAT other side)

7. FUNCTIONAL REACH FORWARD

Examiner Instructions: Examiner places the ruler at the end of the fingertips when the arms are out at 90 degrees. The patient may not lift heels, rotate trunk, or protract scapula excessively. Patient must keep their arms parallel to ruler and may use less involved arm. The recorded measure is the maximum horizontal distance reached by the patient. Record best reach.

Patient: Stand normally. Please lift both arms straight in front of you, with fingertips held even. Stretch your fingers and reach forward as far as you can. Don't lift your heels. Don't touch the ruler or the wall. Once you've reached as far forward as you can, please return to a normal standing position. I will ask you to do this two times. Reach as far as you can.

8. FUNCTIONAL REACH LATERAL

Examiner Instructions: Have subject align feet evenly so that the fingertips, when the arm is out at 90 degrees is at the start of the ruler. The recorded measure is the maximum horizontal distance reached by the patient. Record the best reach. Make sure the subject starts in neutral. The patient is allowed to lift one heel off the floor but not the entire foot.

Patient: Stand normally with feet shoulder width apart. Arms at your sides. Lift your arm out to the side. Your fingers should not touch the ruler. Stretch your fingers and reach out as far as you can. Do not lift your toes off the floor. Reach as far as you can. (REPEAT other side)

TRANSITIONS – ANTICIPATORY POSTURAL ADJUSTMENT

9. SIT TO STAND

Examiner Instructions: Note the initiation of the movement, and the use of hands on the arms of the chair or their thighs or thrusts arms forward

Patient: Cross arms across your chest. Try not to use your hands unless you must. Don't let your legs lean against the back of the chair when you stand. Please stand up now.

10. RISE TO TOES

Examiner Instructions: Allow the patient to try it twice. Record the best score. (If you suspect that subject is using less than their full height, ask them to rise up while holding the examiners' hands.) Make sure subjects look at a target 4-12 feet away.

Patient: Place your feet shoulder width apart. Place your hands on your hips. Try to rise as high as you can onto your toes. I'll count out loud to 3 seconds. Try to hold this pose for at least 3 seconds. Look straight ahead. Rise now.

11. STAND ON ONE LEG

Examiner Instructions: Allow the patient two attempts and record the best. Record the sec they can hold posture, up to a maximum of 30 sec. Stop timing when subject moves their hand off hips or puts

Patient: Look straight ahead. Keep your hands on your hips. Bend one leg behind you. Don't touch your raised leg on your other leg. Stay standing on one leg as long as you can. Look straight ahead. Lift

a foot down.

now. **(**REPEAT other side**)**

12. ALTERNATE STAIR TOUCHING

Examiner Instructions: Use standard stair height of 6 inches. Count the number of successful touches and the total time to complete the 8 touches. It's permissible for subjects to look at their feet.

Patient: Place your hands on your hips. Touch the ball of each foot alternately on the top of the stair. Continue until each foot touches the stair four times (8 total taps). I'll be timing how quickly you can do this. Begin now.

13. STANDING ARM RAISE

Examiner Instructions: Use 2.5 Kg (5 lb) weight. Have subjects stand and lift weight with both hands to shoulder height. Subjects should perform this as fast as they can. Lower score by 1 category if weight must be less than 2.5 Kg (5 lb) +/or lifts < 75 deg.

Patient: Lift this weight with both hands from a position in front of you to shoulder level. Please do this as fast as you can. Keep your elbows straight when you lift and hold. Hold for my count of 3. Begin now.

REACTIVE POSTURAL RESPONSES

14. IN PLACE RESPONSE- FORWARD

Examiner Instructions: Stand in front of the patient, place one hand on each shoulder and lightly push the patient backward until their anterior ankle muscles contract, (and toes just start to extend) then suddenly release. Do not allow any pre-leaning by patient. Score only the best of 2 responses if the patient is unprepared or you pushed too hard.

Patient: For the next few tests, I'm going to push against you to test your balance reaction. Stand in your normal posture with your feet shoulder width apart, arms at your sides. Do not allow my hands to push you backward. When I let go, keep your balance without taking a step

15. IN PLACE RESPONSE- BACKWARD

Examiner Instructions: Stand behind patient, place one hand on each scapula and isometrically hold against patient's backward push, until heels are about to be lifted, not allowing trunk motion. Suddenly release. Do not allow any pre-leaning by patient. Score the best of 2 responses if patient is unprepared, or you pushed too hard.

Patient*:* Stand with your feet shoulder width apart, arms at your sides. Do not allow my hands to push you forward. When I let go, keep your balance without taking a step

16. COMPENSATORY STEPPING CORRECTION-FORWARD

Examiner Instructions: Stand in front to the side of patient with one hand on each shoulder and ask them to push forward. (Make sure there is room for them to step forward). Require them to lean until their shoulders and hips are in front of their toes. Suddenly release your support when the subject is in place. The test must elicit a step. Be prepared to catch patient.

Patient: Stand with your feet shoulder width apart, arms at your sides. Lean forward against my hands beyond your forward limits. When I let go, do whatever is necessary, including taking a step, to avoid a fall.

17. COMPENSATORY STEPPING CORRECTION - BACKWARD

Examiner Instructions: Stand in back and to the side of the patient with one hand on each scapula and ask them to lean backward. (Make sure there is room for them to step backward.) Require them to lean until their shoulders and hips are in back of their heels. Release your support when the subject is in place.

Patient: Stand with your feet shoulder width apart, arms down at your sides. Lean backward against my hands beyond your backward limits. When I let go, do whatever is necessary, including taking a step, to avoid a fall.

Test must elicit a step.

NOTE: Be prepared to catch patient.

18. COMPENSATORY STEPPING CORRECTION- LATERAL

Examiner Instructions: Stand behind the patient, place one hand on either the right (or left) side of the pelvis, and ask them to lean their whole vertial body into your hand. Require them to lean until the midline of pelvis is over the right (or left) foot and then suddenly release your support.

Patient: Stand with your feet together, arms down at your sides. Lean into my hand beyond your sideways limit. When I let go, step if you need to, to avoid a fall.

NOTE: Be prepared to catch patient.

SENSORY ORIENTATION

19. SENSORY INTEGRATION FOR BALANCE (MODIFIED CTSIB)

Examiner Instructions: Do the tests in order. Record the time the patient was able to stand in each condition to a maximum of 30 seconds. Repeat condition if not able to stand for 30 s and record both trials (average for category). Use medium density Temper® foam, 4 inches thick. Assist subject in stepping onto foam. Have the subject step off the foam between trials. Include leaning or hip strategy during a trial as "instability."

Patient*:* For the next 4 assessments, you'll either be standing on this foam or on the normal ground, with your eyes open or closed. Place your hands on your hips. Place your feet together until almost touching. Look straight ahead. Each time, stay as stable as possible until I say stop.

20. INCLINE EYES CLOSED

Examiner Instructions: Aid the patient onto the ramp. Once the patient closes their eyes, begin timing. Repeat condition if not able to stand for 30 s and average both trials/ Note if sway is greater than when standing on level surface with eyes closed (Item 15B) or if poor alignment to vertical. Assist includes use of a cane or light touch any time during the trial.

Patient*:* Please stand on the incline ramp with your toes toward the top. Place your feet shoulder width apart. Place your hands on your hips. I will start timing when you close your eyes.

STABILITY IN GAIT

21. GAIT – LEVEL SURFACE

Examiner Instructions: Place two markers 20 feet (6 meters) apart and visible to the patient on a level walkway. Use a stopwatch to time gait duration. Have subjects start with their toes on the mark. Start timing with the stopwatch when the first foot leaves the ground and stop timing when both feet stop beyond the next mark.

Patient: Walk at your normal speed from here past the next mark and stop.

22. CHANGE IN SPEED

Examiner Instructions: Allow the patient to take 2-3 steps at their normal speed, and then say "fast", after 2-3 fast steps, say "slow". Allow 2-3 slow steps before they stop walking.

Patient: Begin walking at your normal speed, when I tell you "fast" walk as fast as you can. When I say "slow", walk very slowly.

23. WALK WITH HEAD TURNS – HORIZONTAL

Examiner Instructions: Ask the patient to turn their head and hold it so they are looking over their shoulder until you tell them to look over the opposite

Patient: Begin walking at your normal speed, when I say "right", turn your head and look to the right. When I say "left" turn your head and look to the left. Try to

shoulder every 2-3 steps. If the patient has cervical restrictions allow combined head and trunk movements (enbloc).

keep yourself walking in a straight line.

24. Walk With Pivot Turns

Examiner Instructions: Demonstrate a pivot turn. Once the patient is walking at normal speed, say “turn and stop.” Count the steps from turn until the subject is stable. Instability is indicated by wide stance width, extra stepping or trunk and arm motion.

Patient: Begin walking at your normal speed. When I tell you to “turn and stop”, turn as quickly as you can to face the opposite direction and stop. After the turn, your feet should be close together.

25. Step over obstacle

Examiner Instructions: Place the 2 stacked boxes (9” or 22.9 cm height) 10 ft. away from where the patient will begin walking. Use a stopwatch to time gait duration to calculate average velocity by dividing the number of seconds into 20 feet. Look for hesitation, short steps and touch on obstacle.

Patient: Begin walking at your normal speed. When you come to the shoe boxes, step over them, not around them and keep walking.

26. Timed “Get Up & Go”

Examiner Instructions: Have the patient sit with their backs against the chair. Time the patient from the time you say “go” until they return to sitting in chair. Stop timing when the patient’s buttocks hit the chair bottom. The chair should be firm with arms to push from if necessary. TOOLS: TAPE ON FLOOR 3 METERS FROM THE FRONT OF THE CHAIR LEGS**.**

Patient: When I say “GO,” stand up from the chair, walk at your normal speed across the tape on the floor, turn around, and come back to sit in the chair. I will time how long it takes.

27. Timed “Get Up & Go” With Dual Task

Examiner Instructions*:* Before beginning, practice with the patient how to count backward from a number between 90 and 100 by 3s, to make sure they can do the cognitive task. Then ask them to count backwards from a different number and after a few numbers say GO for the GET UP AND GO TASK. Time the patient from when you say “go” until they return to sitting. Stop timing when the patient’s buttocks touch the chair bottom. The chair should be firm with arms to push from if necessary.

Patient: a) Count backwards by 3’s starting at 100 OR b) List random numbers and when I say “GO,” stand up from the chair, walk at your normal speed across the tape on the floor, turn around, and come back to sit in the chair but continue listing numbers**.**
